# Congenital Cataract: Prevalence and Surgery Age at Zhongshan Ophthalmic Center (ZOC)

**DOI:** 10.1371/journal.pone.0101781

**Published:** 2014-07-03

**Authors:** Haotian Lin, Ye Yang, Jingjing Chen, Xiaojian Zhong, Zhaochuan Liu, Zhuoling Lin, Wan Chen, Lixia Luo, Bo Qu, Xinyu Zhang, Danying Zheng, Jiao Zhan, Hanfu Wu, Zhirong Wang, Yu Geng, Wu Xiang, Weirong Chen, Yizhi Liu, and CCPMOH Study Group

**Affiliations:** State Key Laboratory of Ophthalmology, Zhongshan Ophthalmic Center, Sun Yat-sen University, Guangzhou, China; Zhongshan Ophthalmic Center, China

## Abstract

Congenital cataract (CC) is the primary cause of treatable childhood blindness. Population-based assessments of prevalence and surgery age of CC, which are critical for improving management strategies, have been unavailable in China until now. We conducted a hospital-based, cross-sectional study of the hospital charts of CC patients younger than 18 years old from January 2005 to December 2010 at Zhongshan Ophthalmic Center (ZOC) in Guangzhou, China. Residence, gender, age at surgery, hospitalization time, and the presence of other ocular abnormalities were extracted and statistically analyzed in different subgroups. The search identified 1314 patients diagnosed with CC from a total of 136154 hospitalizations, which accounted for 2.39% of all the cataract in-patients and 1.06% of the total in-patients over the six-year study period. Of the identified CC patients, 9.2% had ≥2 hospitalizations due to the necessity of additional surgeries, with a total ratio of boys to girls of 1.75∶1. Based on a subgroup analysis according to age, patients 2–6 years old constituted the highest proportion (29.22%) of all hospitalized CC patients, and those 13–18 years old constituted the lowest proportion (13.47%) of the total number. The average age at surgery was 27.62±23.36 months, but CC patients ≤6 years old (especially ≤6 months old) became increasingly prevalent throughout the 6-year study period. A total of 276 cases (20.93%) of CC were associated with one or more other ocular abnormalities, the highest incidence rates were observed for exotropia (6.24%), nystagmus (6.16%), and refractive error (3.65%). In conclusion, CC patients accounted for 2.39% of all cataract in-patients in a review of 6 years of hospitalization charts from ZOC. The age at the time of surgery decreased over the 6-year study period, which probably reflects the continuing improvement of public awareness of children’s eye care in China.

## Introduction

Congenital cataract (CC) is among the primary causes of treatable childhood blindness, [1] and its incidence varies in different reports. The global prevalence of congenital cataract is 1 to 15 per 10,000 children; [2] in the United States, the incidence is 2.0 per 10,000 births [3], [4]. In China, the incidence of CC is approximately 5.0 per 10,000 births [5] [6], and 22% to 30% of childhood blindness [7] is attributed to CC in the absence of appropriate treatment, with delayed presentation to hospitals and late surgical treatments found to be the major causes of blindness and visual impairment. [8] A national prevention and treatment program is crucial for the early diagnosis and timely treatment of CC. [9] However, to our knowledge, published reports of population-based assessments of the prevalence and incidence of CC in China has been unavailable until now.

Data on epidemiology and surgery age are critical for improving management strategies for this challenging condition. Many of the available studies that have examined variables other than overall aggregate incidence have been restricted to small numbers of patients. [10],[11] Unlike senile cataract, CC responds poorly to medical therapy, and successful management depends primarily on early diagnosis and referral for surgery when indicated; [12] as a result, surgery is the first-line treatment, and the age at surgery is a critical success factor. CC treatment at Zhongshan Ophthalmic Center (ZOC), as China’s first and one of the best and largest eye hospitals [13], is representative of the current CC treatment methods in southern China. In the Medical Records Department of ZOC in Guangzhou, we were able to review a large representative sample of hospitalized cataract patients who underwent surgery over a 6-year period in order to better assess the epidemiology of and surgery age for CC. The epidemiology of hospitalized CC patients could perhaps reflect its prevalence in the broader population.

## Methods

### Ethics Statement and Search Strategy

This study was designed as a hospital-based, cross-sectional study. The study was included in the Childhood Cataract Program of the Chinese Ministry of Health (CCPMOH) [14]–[17] and was approved by the institutional review board of Zhongshan Ophthalmic Center in Sun Yat-sen University (IRB-ZOC-SYSU), Guangzhou, China. The tenets of the Declaration of Helsinki were followed throughout this study. Patient records/information was anonymized and de-identified prior to analysis, and our study was exempted from participant consent by the institutional review board of Zhongshan Ophthalmic Center in Sun Yat-sen University (IRB-ZOC-SYSU). CC patients younger than 18 years who were treated from January 1, 2005, to December 31, 2010, at ZOC in China were the search targets. In the database of the Medical Records Department of ZOC, cataract types and related conditions were coded using the International Classification of Diseases, Ninth Revision, Clinical Modification (ICD-9-CM).[18] The following codes were utilized for case identification in the records: infantile cataract (366.0), congenital cataract and lens anomalies (743.3), and aphakia and other disorders of the lens (379.3).

### Inclusion Criteria

We identified pediatric patients with the above three codes and created an eligible database with CC patients ≤18 years old. Most of the diagnoses were accepted as recorded in the charts. Individual case records were reviewed to confirm the presence of CCs, without differentiation of inherited or sporadic anomalies but with differentiation of associated ocular anomalies. [19] The surgeries included cataract surgery and/or intraocular lens (IOL) implantation, treatment of the sequelae of CC, or management of associated ocular abnormalities.

### Information Extraction

All of the eligible charts were carefully reviewed by two independent researchers. We recorded the incidence of CC, gender, presenting age of each patient, laterality, and associated ocular abnormalities, as well as the type and number of surgical procedures. The prevalence of strabismus was recorded without differentiating between development pre- and post-cataract surgery. Because of the retrospective nature of our study, it was not always clear whether the strabismus noted was present preoperatively or postoperatively. Furthermore, more than half of the records did not contain accurate visual acuity recordings because the children were too young for visual acuity assessments, and many of the records only contained information on fixation preference. Therefore, we did not include vision measurements in this analysis. We also did not include information on pharmacotherapy. Above all, age at surgery, gender, hospitalization time, and the presence of other ocular abnormalities were considered to be reliable and accurate information and were extracted for different subgroups. For analysis and reporting in this study, we divided our cohort into 5 groups according to age as follows: less than 6 months old (≤6 M), 6 months to 2 years old (6 M< and ≤2 Y), 2 years old to 6 years old (2 Y< and ≤6 Y), 6 years old to 12 years old (6 Y< and ≤12 Y), and 12 years old to 18 years old (12 Y< and ≤18 Y). Furthermore, to assess the trends in CC treatment, our cohort was also divided into 3 groups according to hospitalization time: hospitalization from 2005 to 2006, hospitalization from 2007 to 2008, and hospitalization from 2009 to 2010. For the purpose of this article only (and not for the data analysis), data regarding duration were rounded to the nearest integer for any duration longer than 1 month.

### Statistical Analysis

All of the data were entered into a Microsoft Excel (Microsoft Corp., Redmond, Washington, USA) spreadsheet, classified and analyzed by two of the authors, and mutually checked. The data were also entered into SPSS statistical software, version 17.0 for Windows (SPSS Inc., Chicago, Illinois, USA) for statistical analysis. The χ^2^ test and Fisher’s exact test were used for categorical variables, with P<0.05 was considered statistically significant.

## Results

During the period of this cross-sectional study, there were a total of 136154 hospitalizations at ZOC for different eye diseases. Among these cases, 60188 (44.2%) hospitalizations were for cataract surgeries, and only 1439 (1.06%) hospitalizations were for CC surgeries, with a total of 1314 CC patients identified. Most of the CC patients (1193/1314, 90.8%) underwent only one surgery during one hospitalization; however, 117 (8.9%) CC patients underwent two surgeries during two hospitalizations and 4 (0.3%) CC patients underwent 3 surgeries during 3 hospitalizations. Of these patients, 24.5% (322/1314) had unilateral cataracts. The right and left eyes were involved in 11.5% (151/1314) and 13.1% (171/1314) of cases, respectively, with the remaining 75.5% (992/1314) of cases consisting of bilateral involvement.

**Figure 1:**
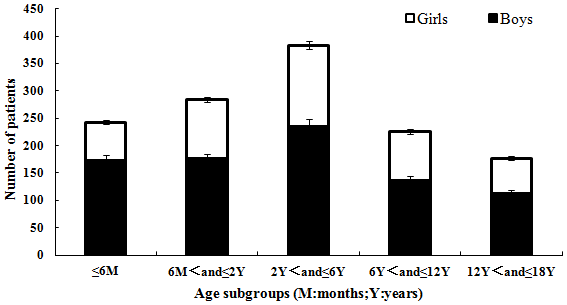
Analysis of the number of patients in and gender ratio of each age subgroup. M = months, Y = years.

According to the demographic analysis, all 1314 of the CC patients identified were from the southern region of China, with boys comprising 63.6% of this patient population (ratio of boys to girls, 1.75∶1). According to the age subgroup analysis, patients 2–6 years old constituted the highest proportion (29.22%) of all hospitalized CC patients, and those 13–18 years old constituted the lowest proportion (13.47%) of the total number. However, there were no significant differences (P = 0.064) in the gender ratio between the different age subgroups, as shown in Figure 1.

The prevalence rates of CC among ZOC patients during the study period ranged from 1.01% to 1.10%, with no significant differences between the included years. However, the total number of hospitalizations for cataract surgeries (including senile cataracts) increased during the study period (data not shown).

**Figure 2:**
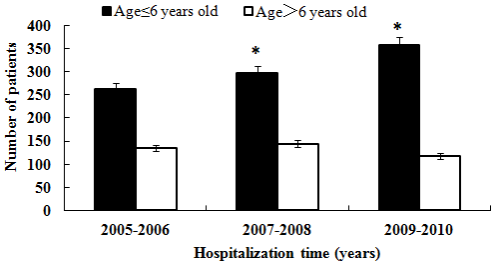
Numbers of hospitalized patients in the age subgroups (age ≤6 years old vs. >6 years old) in different years (P<0.05).

**Figure 3:**
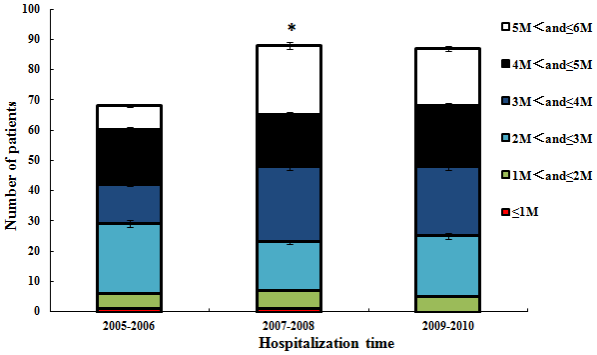
Total numbers of hospitalized patients ≤6 months old in different years (P<0.05).

The average age at surgery was 27.62±23.36 months, with no significant differences (P>0.05) between the hospitalization year subgroups. We also conducted an analysis of the trend in age at CC presentation and found that there was a significantly increasing trend in the population presenting at age ≤6 years over the study period, as shown in Figure 2. In particular, a presenting age of <6 months was considered to be associated with a better prognosis after CC surgery, and we found that the number of infants (5 M< and ≤6 M) who underwent surgery during this critical period increased beginning in 2007, as shown in Figure 3.

**Figure 4:**
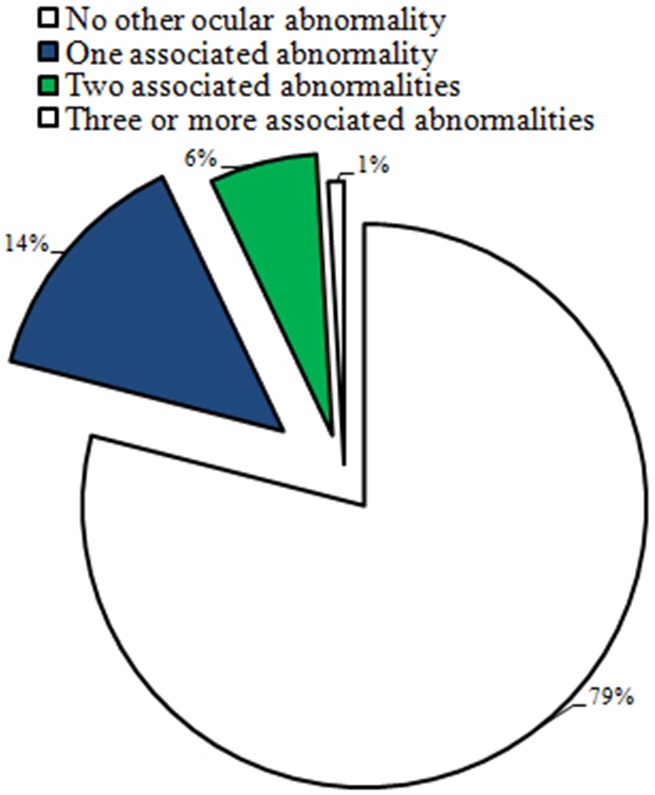
Distribution of patients with other ocular abnormalities.

It is well accepted that the occurrence of other isolated ocular anomalies makes a large difference in the prognosis of CC. [20],[21] In our study, we identified 1039 (79.1%) cases with no records of any other ocular abnormalities and a total of 275 (21.0%) cases with a record of one or more other ocular abnormalities, as shown in Figure 4.

Different associated ocular abnormalities also contribute differently to prognosis. [22]–[24] Details regarding the associated ocular abnormalities in the study population are shown in Table 1. The prevalence of strabismus (exotropia+esotropia) in our study was 9.5% (124/1314). When comparing the presence of strabismus in patients with unilateral versus bilateral cataracts, it was found that almost three-quarters of the patients (72.6% [90/124]) with strabismus had unilateral cataracts (P<0.001). Of the cases presenting with strabismus preoperatively or postoperatively, 6.5% (8/124) had persistent hyperplastic primary vitreous (PHPV) or congenital persistent pupillary membrane (CPPM). The prevalence of nystagmus was 6.16% (81/1314), which was the second most common ocular anomaly in our study. Refractive error (3.65% [48/1314]), congenital microcornea (0.99% [13/1314]), and congenital microophthalmia (0.68% [9/1314]) were the next three most common ocular abnormalities diagnosed during hospitalization for CC in our study. Other than the ocular anomalies mentioned above, when specific disease patterns could not be identified, isolated central nervous system anomalies, such as microcephaly, ventriculomegaly, and sensorineural hearing loss, were found to be most frequently associated with cataracts.

**Table 1:** Associated ocular abnormalities and their incidences.

Associated abnormality	Number of cases	Incidence rate(%)
Exotropia	82	6.24
Nystagmus	81	6.16
Refractive error	48	3.65
Esotropia	42	3.20
Congenital microcornea	13	0.99
Congenital microphthalmia	9	0.68
PHPV*	5	0.38
Congenital aniridia	5	0.38
CPPM#	3	0.23
Congenital ptosis	2	0.15

*PHPV = Persistent hyperplastic primary vitreous.

# CPPM = Congenital persistent pupillary membrane.

## Discussion

Cataract is among the leading causes of blindness world-wide. [25] Additionally, although CC constitutes only a small proportion of these cases, it is one of the primary causes of treatable childhood blindness. [1], [2] Data on epidemiology and surgery age are critical for improving management strategies for this challenging condition, but many of the available studies that have examined variables other than overall aggregate incidence have been restricted to small numbers of patients. [3]–[6] In this study, we performed a hospital-based, cross-sectional investigation consisting of the review of ZOC hospitalization charts over a 6-year period and found that CC patients accounted for 2.39% of the total cataract in-patient population. Additionally, a higher incidence of CC was found among male patients, and more than one-fifth of the cases were associated with other ocular abnormalities. The average age at surgery was 27.62±23.36 months, with no significant difference (P>0.05) between different year subgroups but with a trend toward younger ages over the 6-year period. Our study may reflect the prevalence of CC in the broader population, and it could provide a useful reference for the development of medical science policies for the advocacy for and prevention of this cause of childhood blindness, before any national epidemiological data are available [5].

To our knowledge, published reports on population-based assessments of the prevalence and incidence of CC have been unavailable until now. Some available small-population studies have shown that the incidence of CC is approximately 5.0 per 10,000 births in China. [7], [8] In our large-population, hospital-based study, we found that CC patients accounted for 2.39% of the total cataract in-patients. This rate does not necessarily reflect the national prevalence rate, but it at least partially reflects the prevalence of this blinding disease in Chinese children. Moreover, it remains unknown whether there is a gender difference in the incidence of CC. [6] In the present study, we found that the ratio of boys to girls among the children with CC was 1.75∶1, which was greater than the Chinese population gender ratio (male: female = 51.27∶48.73%), according to the sixth national census data. The high proportion of boys with CC may also be attributed to traditional son preferences in China or to gender-related genetic mechanisms, which remain unknown and should be investigated in future studies [17].

Unlike senile cataract, CC responds poorly to medical therapy, and successful management is dependent on early diagnosis and referral for surgery when indicated; thus, surgery is the first-line treatment, and the age at surgery is a critical success factor [12]. Currently, CC treatment is most successful during the critical period of visual development, with surgical and post-surgical treatments for refractive error correction being performed as early as possible to prevent patients from developing the irreversible form of deprivation amblyopia. [20] Therefore, early diagnosis, choosing the appropriate time for surgery, improved surgical techniques, prevention of postoperative refractive amblyopia, and timely training are very important for the recovery of postoperative visual function. Because CC screening in many developed countries was established as early as the 1990s, [20], [26], [27] 64% to 78% of children with CC can be identified within 100 days after birth. Some cases can even be identified in the first month after birth. However, in developing countries, the patient’s age at the time of CC surgery is generally 2–4 months old or older. [28] In China, which has no national screening or follow-up system for CC, the surgery age for CC remains unclear. In the present study, the average age at surgery was 27.62±23.36 months, and patients 2–6 years old constituted the highest proportion (29.22%) of the study population. Only 18 and 243 patients were younger than three months old and 1–6 months old, respectively, accounting for 18.49% of the total patient population. Our findings demonstrated that delayed presentation to the hospital and late surgical treatment are very common in China, which may have been the major reasons for the poor prognosis associated with CC, consistent with previous reports. However, there was another more interesting and important finding in our study. The number of CC patients ≤6 years old (especially ≤6 months old) increased over the 6-year study period. This trend may be attributed to the strengthened recognition of the importance of early treatment for CC by parents and pediatric ophthalmologists and to the continuing improvement of ophthalmic surgical techniques and anesthetic techniques in China [13], [14].

Although the timing of surgery is critical for CC prognosis, it has also been reported that the presence of other associated ocular abnormalities, [19], [23], [29] such as nystagmus, strabismus, PHPV, CPPM, small eyes, and small corneal disorders, also has a significant effect on prognosis. In this study, a total of 275 patients had associated ocular abnormalities, accounting for 1/5 of all the patients. It has been reported that preoperatively identified nystagmus indicates impending amblyopia or other types of visual impairment [30]. In our study, 81 patients were preoperatively identified as having nystagmus, constituting 6.16% of the total number of cases, which indicates that the postoperative recovery of visual function in these patients would have been poor due to delayed surgery.

Several limitations of our study should be considered when interpreting the results. First, the data do not provide a complete representation of the population but only of surgical patients at the best eye center in China, with admissions biased toward patients with complicated or serious diseases. Second, our research did not include data on visual outcomes. Third, the patients may have changed hospitals or visited other hospitals, so the exact number and type of surgical procedures performed on the study population are unknown. Despite these limitations, the results of this study provide useful information on the epidemiology of CC due to the lack of available large-population-based investigations in China. Future population-based surveys and prospective clinical studies focused on CC should be performed. Although large population-based or even national surveys of the incidence rate of CC would be the best way to initiate the development of a national prevention and treatment program, such surveys would be time-consuming and costly.

In summary, this study investigated the prevalence, gender, age at surgery, and associated ocular abnormalities of CC patients among a large cohort of surgical patients at ZOC, which is China’s first and one of China’s best and largest eye hospitals. The findings of our study could provide a useful foundation for the advocacy for and prevention of this cause of childhood blindness.
